# Sub‐dose anesthetics combined with chloride regulators protect the brain against chronic ischemia–hypoxia injury

**DOI:** 10.1111/cns.14379

**Published:** 2023-08-06

**Authors:** Chenyi Yang, Ye Wang, Yun Li, Xinyi Wang, Wei Hua, Zhuo Yang, Haiyun Wang

**Affiliations:** ^1^ Nankai University Tianjin China; ^2^ Nankai University Affinity the Third Central Hospital Tianjin China; ^3^ The Third Central Clinical College of Tianjin Medical University Tianjin China; ^4^ Tianjin Key Laboratory of Extracorporeal Life Support for Critical Diseases Tianjin China; ^5^ Artificial Cell Engineering Technology Research Center Tianjin China; ^6^ Tianjin Institute of Hepatobiliary Disease Tianjin China

**Keywords:** chloride ion, cognitive function, hypoxia, ischemia, propofol, sevoflurane, γ‐Aminobutyric acid

## Abstract

**Background:**

Cerebral ischemia–hypoxia leads to excitotoxicity‐mediated neuronal damage and cognitive dysfunction, especially in the elderly. Excessive intracellular [Cl^−^]_i_ accumulation weakens γ‐aminobutyric acid (GABA) compensatory effects. Sub‐anesthetic dose of propofol protected the brain against ischemia–hypoxia, which was abolished by blocking Cl^−^ efflux transporter K^+^/Cl^−^ cotransporter 2 (KCC2). We aimed to determine whether low‐dose anesthetic combined with [Cl^−^]_i_ regulators could restore the compensatory GABAergic system and improve cognitive function.

**Methods:**

Chronic cerebral hypoxia (CCH) model was established by bilateral carotid artery ligation in aged rats. Sub‐dose of anesthetics (propofol and sevoflurane) with or without KCC2 agonist N‐ethylmaleimide (NEM) or Na^+^/K^+^/Cl^−^ cotransporter 1 (NKCC1) antagonist bumetanide (BTN) was administered systemically 30 days post‐surgery. Primary rat hippocampal neuronal cultures were subjected to hypoxic injury with or without drug treatment. Memory function, hippocampal neuronal survival, GABAergic system functioning, and brain‐derived neurotrophic factor (BDNF) expressions were evaluated.

**Results:**

Sub‐anesthetic dose of combined propofol (1.2 μg mL^−1^) and sevoflurane [0.7 MAC (minimum alveolar concentration)] did not aggravate the hypoxic brain injury in rats or cell damage in neuronal cultures. Adding either BTN or NEM protected against hypoxic injury, associated with improved cognitive function in vivo, less intracellular accumulation of [Cl^−^]_i_, reduced cell death, restored GABAergic compensation, and increased BDNF expression both in vivo and in vitro.

**Conclusion:**

Sub‐anesthetic dose of propofol and sevoflurane is a recommended anesthesia regimen in at‐risk patients. Restoration of [Cl^−^]_i_ homeostasis and GABAergic could further reduce the brain damage caused by ischemia–hypoxia.

## Graphical abstract

The sub‐anesthetic doses of propofol and sevoflurane in combination with [Cl^−^]_i_ transporter modulator bumetanide or N‐ethylmaleimide can reduce cognitive impairment caused by ischemia–hypoxia by restoring BDNF expression and GABAergic system.

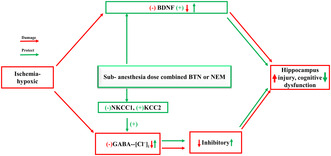

## 1 INTRODUCTION

Vascular cognitive impairment and dementia (VCID) is the second most common type of cognitive impairment, only behind Alzheimer's disease. How to prevent the progression of VCID is an important focus at present. Chronic cerebral ischemia (CCH) is the major pathology of VCID and has been shown to cause hippocampal and white matter damage, hemorrhage, brain atrophy and memory impairment.^1^ Studies have found that ischemic–hypoxic brain injury occurs when the cerebral perfusion drops to 35–50 mL 100 g^−1^ min^−1^, which means that the blood oxygen saturation may be lower than 90%.^2^ In this context, performing anesthesia in elderly VCID patients is particularly challenging due to significant higher risk of peri‐operative cognitive deterioration compared to general population.

Compared with other regions, the hippocampal CA1 region is most vulnerable to ischemia–hypoxic injury.^3^ Excitotoxicity is a major cause of neuronal death in ischemia–hypoxic injury, which can be compensated by inhibition of the neuronal activation through γ‐aminobutyric acid (GABA)‐mediated Cl^−^ flow.^4^ Transporters regulating Cl^−^ homeostasis include the K^+^/Cl^−^ co‐transporter KCC2, responsible for the efflux of Cl^−^ out of the cell,^5^ and the Na^+^/K^+^/Cl^−^ co‐transporter NKCC1, responsible for the influx of Cl^−^ into the cell.^6^ Indeed, these two transporters are involved in age‐related anesthesia neurotoxicity upon isoflurane exposure.^7^ Both preclinical and clinical studies have shown that the regulation of the GABAergic system may counteract excitation/inhibition (E/I) imbalance and improve diseases outcome.^8^ The inhibitory effect of GABA depends on the hyperpolarization mediated by Cl^−^ influx through the GABA_A_ receptor (GABA_A_R) channel, making intracellular [Cl^−^]_i_ the main determinant of GABA's inhibitory effect and a key factor in various brain pathologies.^9^ Made of a subunit repertoire, GABA_A_R is closely related to cognitive and memory functions.^10^ For example, a significant reduction in GABA_A_R α1 subunit was observed in rat hippocampal CA1 neurons in chronic ischemic encephalopathy,^11^ and L‐655, the inverse agonist of GABA_A_R α5, could reverse the effect of isoflurane‐induced memory impairment in elderly rats.^12^

Sub‐anesthetic dose combined propofol and sevoflurane (PS) has emerged as a reliable strategy in the elderly.^13^ Sevoflurane has been used more extensively than isoflurane in recent years,^14^ although its effect on cognition is underexplored. Interestingly, both KCC2 antagonist (dihydroindenyl) alkanoic acid and KCC2 upstream control protein inhibitor PKMζ reversed the protective effect of sub‐anesthetic dose of propofol on ischemic neurons,^15^, ^16^ suggesting a critical role of [Cl^−^]_i_ homeostasis. In the present study, we test the effect of combining low dose of PS with Cl^−^ regulators in a mouse VCID model.

Brain‐derived neurotrophic factor (BDNF) is the most abundant neurotrophin in the brain, whose expression is significantly decreased by ischemia and hypoxia,^17^ therefore could be applied to indicate the extent of injury severity. It could also attenuate excitotoxicity by inhibiting calcium release.^18^ We here report that low‐dose PS did not affect CCH‐induced injury. However, adding either an NKCC1 blocker [bumetanide (BTN)]^19^ or a KCC2 agonist [N‐ethylmaleimide (NEM)]^20^ can salvage the damage and improve neurocognitive outcome, suggesting the potential for perioperative regulation of NKCC1/KCC2‐[Cl^−^]_i_‐GABA_A_R pathway as an anesthetic strategy in VCID patients.

## 2 METHODS

### 2.1 Primary hippocampal neuronal culture and hypoxia

Primary hippocampal neuronal cultures were performed as previously described.^21^ In brief, brains from neonatal rats (postnatal day 1–3) were collected and meninges were peeled off. Hippocampus was dissected and vascular plexus was carefully removed under a dissection microscope. Tissues were then dissociated with trypsin (0.25%) and DNase (200 μg mL^−1^) and cell suspension was plated in Neurobasal‐A medium (Gibco, Thermo Fisher Scientific, Waltham, MA, USA) supplemented with B27 (Gibco) and antibiotics (penicillin–streptomycin solution; HyClone) onto a petri dish coated with d‐polylysine (70,000–150,000 kDa, 100 μg mL^−1^; Sigma‐Aldrich) at a density of 1 × 10^5^ cells cm^−2^. Dishes were maintained in a 5% CO_2_ incubator at 37°C and medium was changed every other day up to day 20 to mimic aged neurons. The successful neuronal culture was confirmed by microtubule‐associated protein 2 (MAP2) staining (Figure S1).

To induce hypoxia, neurons were placed in a hypoxic incubator at 37°C with 5% CO_2_, 3% O_2_, and 92% N_2_ for 3 h.^22^ Cells were then placed back in fresh culture medium under normoxia (95% air, 5% CO_2_) for 24 h in the presence or absence of drugs at following concentrations: 4 μg mL^−1^ propofol for full‐dose propofol (P‐high) group, 1.3 MAC sevoflurane for full‐dose sevoflurane (S‐high) group, 1.2 μg mL^−1^ propofol and 0.7 MAC sevoflurane for PS‐low group, BTN 75 μM, and NEM 100 μM.

### 2.2 Cell viability and death assay

Cell viability was determined using a cell counting kit (CCK‐8; Dojindo Laboratories). In brief, 10 μL of CCK‐8 was incubated with neuronal cultures for 2 h at 37°C in the dark, according to the manufacturer's instructions. A microplate reader (ELX800; BioTek) was then used to detect absorbance at 450 nm, which reflects cell viability. The cell viability was calculated as follows: cell viability (%) = (OD_experimental_–OD_blank_)/(OD_CON_–OD_blank_) × 100%.

Cell death was determined by lactic dehydrogenase (LDH) release using the LDH assay kit (Abcam Inc.). In brief, 100 μL LDH reaction mix was added to each well and incubated for 30 min. The absorbance values were measured at 490 nm on the microplate reader (ELX800; BioTek). Results were normalized to the control group, the amount of LDH release of which was considered as 100%.

### 2.3 Intracellular chloride detection

Intracellular [Cl^−^]_i_ levels were detected by the chloride‐ion fluorescent probe N‐(ethoxycarbonylmethyl)‐6‐methoxy‐quinoline bromide (MQAE), whose intracellular fluorescence signal quenches at a velocity proportionate to chloride‐ion concentration.^23^ In brief, the cells were incubated with 10 mM MQAE (Beyotime Biotechnology) at 37°C for 2 h and washed with Krebs‐HEPES buffer for 5 min. The fluorescence intensity of MQAE was detected by flow cytometry.

### 2.4 Animals

The study was designed in accordance with ARRIVE guidelines. All animal procedures were approved by the Animal Research Ethics Committee of Tianjin Medical University (TMUaMEC 2,020,017). Aged male SD rats of 16–18 months old weighing 400 to 550 g were purchased from the Chinese People's Liberation Army Academy of Military Medical Sciences [SCXK (jing) 2019‐0008] and housed with a 12:12 h light–dark cycle at 22–24°C, with ad libitum access to food and water. All the rats were randomly assigned to the following five groups (*n* = 22/group): (1) sham surgery (Sham), (2) CCH, (3) CCH + sub‐anesthetic dose combining propofol and sevoflurane (PS‐low), (4) CCH + PS‐low + BTN (MCE), and (5) CCH + PS‐low + NEM (MCE). The sample size is set based on our previous experience.^13^, ^24^ All the outcome assessments were performed by investigators blinded to the group assignments.

### 2.5 Bilateral carotid artery stenosis (BCAS) and drug administration

In vivo rat VCID models were established by CCH induced by BCAS as previously described.^24^ In brief, rats were anesthetized by intraperitoneal injection of 10% thiobarbital (100 mL kg^−1^). Following a midline neck incision, bilateral common carotid arteries (CCA) were exposed and freed and tightened with a blunt‐tip syringe needle (0.45 mm in diameter, 1 cm in length) at 1.5 cm proximal to the bifurcation of the internal and external carotid arteries. CCA was then ligated with thread at the site of the needle, which was then carefully removed. The wound was sutured and the skin was disinfected.

BTN and NEM were dissolved in saline. Thirty days after BCAS, PS‐low was administered by continuous intravenous injection of propofol at 20 mg kg^−1^ h^−1^ combined with inhalation of sevoflurane at MAC of 0.7 for 3 h. BTN or vehicle was injected intravenously at 15 mg kg^−1^,^25^, ^26^ and NEM or vehicle was injected intraperitoneally at 10 mg kg^−1^.^27^, ^28^ The time points of drug injection were 6 h before anesthesia, during anesthesia, and 6 h after anesthesia. The experiment was carried out the next day after drug administration.

### 2.6 Slice electrophysiology

We used the whole‐cell patch‐clamp technique to record micro‐postsynaptic inhibitory currents (mIPSC) of pyramidal neurons in the hippocampal CA1 region to evaluate the tissue's response to GABA, as previously reported.^29^ In brief, fresh hippocampal slices were placed at room temperature in pre‐gassed (95% O_2_ and 5% CO_2_) artificial cerebrospinal fluid. After establishing a stable whole‐cell configuration for 5–10 min, the Multiclamp 700B amplifier (Molecular Devices) was used to record the mIPSC in the hippocampal CA1 area at −60 mV. Data were discarded if over 20% of change in resistance was observed. All recorded analog signals were filtered at 2 kHz and digitized at 10 kHz using Digidata 1440A and pClamp 10 software (Molecular Device). Clampfit 10 was used to analyze the frequency and amplitude data of the mIPSC.

### 2.7 Contextual fear conditioning test

Contextual fear conditioning test was performed to evaluate the situational memory. The experiment included a training phase and a testing phase. Rats were put into a test chamber. During the training phase on the day of drug administration, a single high‐frequency sound (an auditory cue, 4000 Hz, 80 dB) was applied to the rat for 30 s. In the last 2 s, an electric current of 0.8 mA was applied to the rat's foot. After stimulation, the rat was allowed to continue to explore the test chamber for another 2 min. In the testing phase, 24 h after the training phase, the rat was returned to the same test chamber for 3 min without any sound or foot shock. A video‐tracking system (Stolting) was used to calculate the percentage of time the rat was frozen within the 3 min to estimate contextual fear memory.

### 2.8 Nissl staining

After the behavioral test, the rats were deeply anesthetized and perfused with normal saline and 4% paraformaldehyde solution before cardiac arrest. The brain was collected and fixed with 4% paraformaldehyde, then embedded in paraffin. Coronal sections of hippocampus (3.0 μm) were made and deparaffinized with xylene, dehydrated with gradient alcohol, and then stained with 1% Nissl staining solution (toluidine blue method, Solomen). The stained brain sections were treated again with alcohol and xylene, and sealed with neutral gum. Sections were examined by an observer who was blinded to the experimental conditions under light microscopy at a magnification of 100×/200×. The number of surviving neurons of CA1 area were counted in each section (*n* = 6/group). Only pyramidal neurons showing normal morphology with distinct cytoplasmic and nuclear outlines and a visible nucleolus were counted. Analysis of the data was performed by using Image Pro Plus 6.0 software (Media Cybernetics Co.).

### 2.9 Western blot

A radioimmunoprecipitation assay buffer (RIPA; Solarbio, R0010) supplemented with phenylmethanesulfonyl fluoride (PMSF; Solarbio, P0100) was used to lyse the cultured neuronal cells and hippocampal tissue in the rat brain. In a subset of experiments, Mem‐PER Plus Membrane Protein Extraction Kit (Thermo Fisher Scientific) was used to extract membrane proteins. The protein concentration was determined using bicinchoninic acid (BCA) protein detection kit (Beyotime Biotechnology). After denaturation, equal amount of protein samples was separated by sodium dodecyl sulfate‐polyacrylamide gel electrophoresis (SDS‐PAGE) and transferred to a polyvinylidene fluoride (PVDF) membrane (Millipore, 0.45 μm). After blocking with 5% skimmed milk, membranes were sequentially incubated with primary antibodies [BDNF (1:1000, Cell Signaling Technology, #47808), GABA_A_R α1 (1:1000, Abcam, #ab94585), GABA_A_R α5 (1:1000; Abcam, #ab259880), and GAPDH (1:1000; Abcam, #ab8245)] and secondary antibodies (goat anti‐rabbit IgG H&L, HRP; 1:2000; Abcam, #ab6702). Bands were visualized with an enhanced chemiluminescence detection kit (EMD Millipore). Using Image‐Pro Plus (Media Cybernetics), the band density was analyzed and standardized as fold of GAPDH.

### 2.10 Statistical analysis

GraphPad Prism software (version 9.00; GraphPad Software, La Jolla, CA, USA; www.graphpad.com) was used for all data analyses. The Shapiro–Wilk test was used to check the normality of the data. Data of normal distribution were expressed as mean ± SD. For animal behavior, we used the two‐way analysis of variance (ANOVA), and for other experiments, one‐way ANOVA was used. Tukey's post‐hoc test was performed for all experiments. All experiments were repeated at least three times, and *p* < 0.05 was considered statistically significant.

## 3 RESULTS

### 3.1 Sub‐anesthetic dose of combined PS did not cause further damage to hypoxic neuronal cultures

**FIGURE 1:**
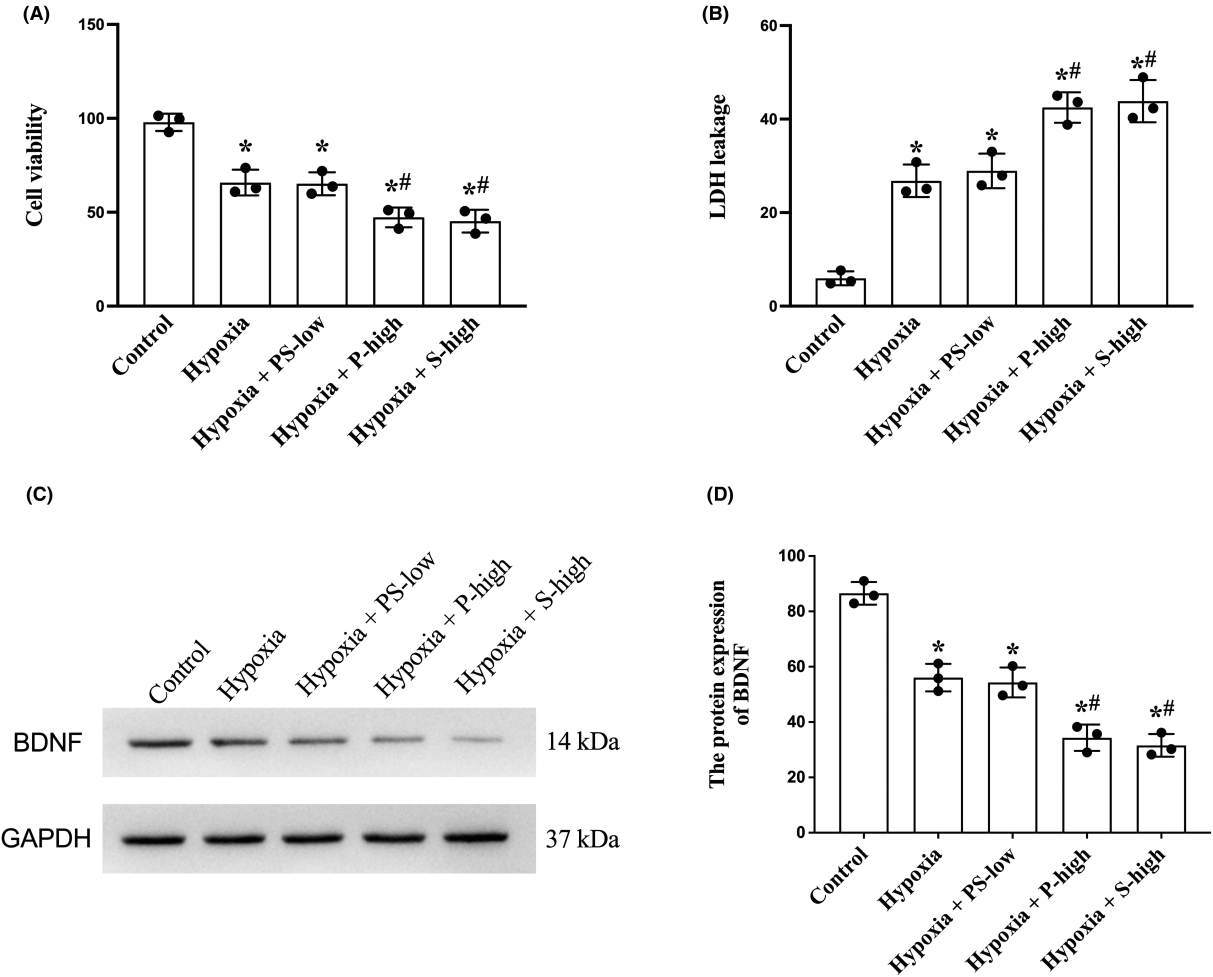
Sub‐anesthetic dose of combined PS did not cause further damage to hypoxic neuronal cultures. Primary hippocampal neuronal cultures were subjected to hypoxia followed by reoxygenation with or without anesthetic treatment. (A) Neuronal viability measured by CCK‐8. (B) Neuronal death measured by LDH release. (C) Western blotting and (D) semi‐quantification of BDNF levels. BDNF, brain‐derived neurotrophic factor; CCK, cell counting kit; LDH, lactic dehydrogenase; P, propofol; PS‐low, sub‐anesthetic dose of combined propofol and sevoflurane; S, sevoflurane. Data are mean ± SD. *p < 0.05 versus control; ^#^ *p* < 0.05 versus hypoxia.

We first determined the effect of sub‐anesthetic dose of combined PS on hypoxic neurons in vitro. CCK‐8 and LDH assays were applied to detect hippocampal neuronal viability and death upon hypoxia and drug treatments (Figure 1A,B). As expected, hypoxia alone significantly decreased cell viability and increased cell death, which was not exacerbated by sub‐dose combined PS. On the contrary, single high‐dose anesthetics caused more significant damages. As for BDNF expression, we found that hypoxia decreased BDNF expression, which was worsened by single high‐dose anesthetics but not by sub‐dose combined anesthetics (Figure 1C,D).

### 3.2 Both BTN and NEM improved hypoxia‐induced [Cl^−^]_i_ dysregulation and survival in neuronal cultures

**FIGURE 2:**
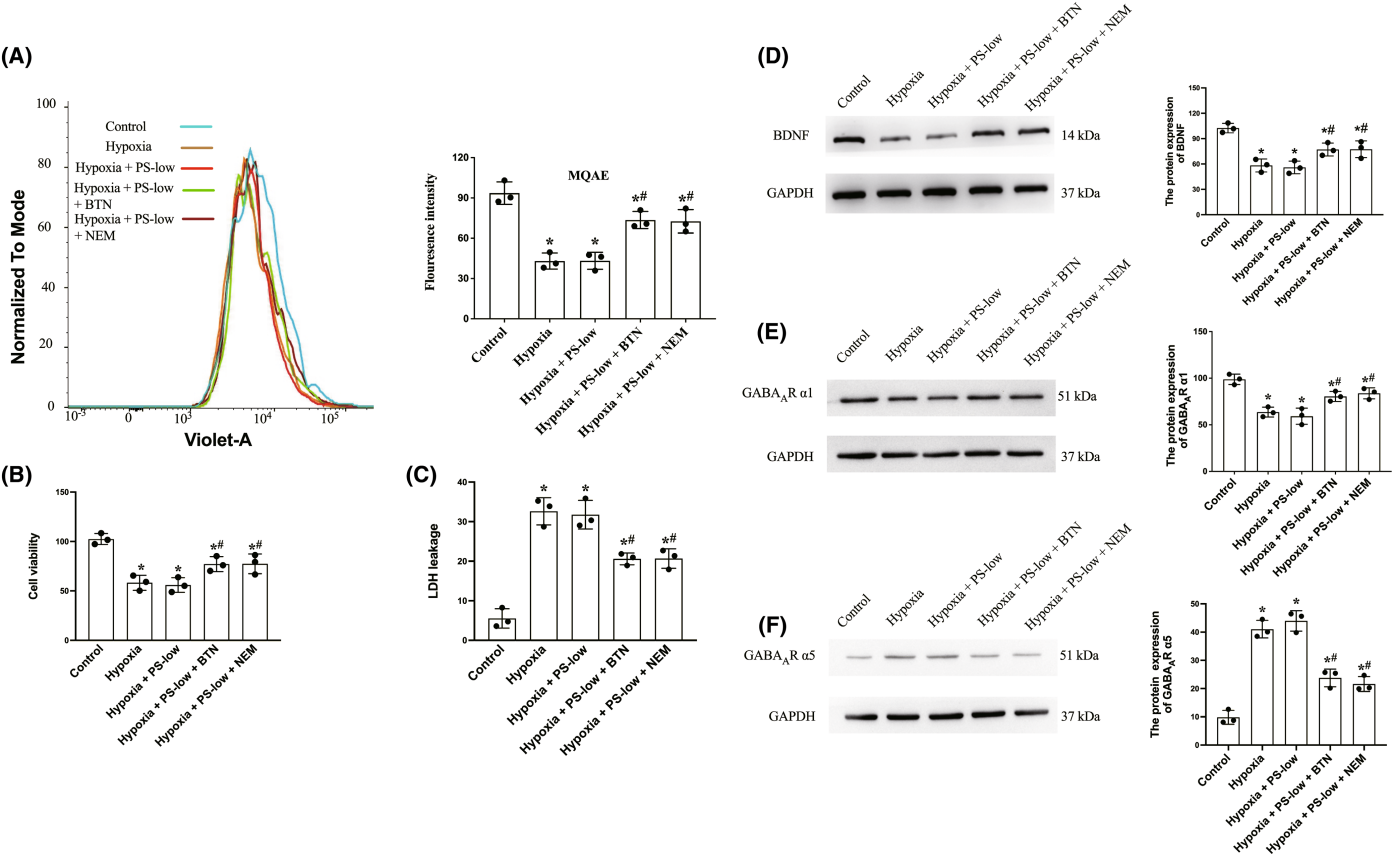
Both BTN and NEM improved hypoxia‐induced [Cl^−^]_i_ dysregulation and survival in neuronal cultures, and also preserved BDNF and GABA_A_R subunit balance. Primary hippocampal neuronal cultures were subjected to hypoxia followed by reoxygenation with or without PS‐low or BTN or NEM. (A) Flow cytometry on intracellular [Cl^−^]_i_ concentration detected by MQAE and quantification. (B) Neuronal viability measured by CCK‐8. (C) Neuronal death measured by LDH release. (D) Western blotting and semi‐quantification of BDNF levels. (E) Western blotting and semi‐quantification of GABA_A_R α1 levels. (F) Western blotting and semi‐quantification of GABA_A_R α5 levels. MQAE: N‐(ethoxycarbonylmethyl)‐6‐methoxy‐quinoline bromide. BDNF, brain‐derived neurotrophic factor; BTN, bumetanide; CCK, cell counting kit; GABA, γ‐aminobutyric acid; LDH, lactic dehydrogenase; NEM, N‐ethylmaleimide; PS‐low, sub‐anesthetic dose of combined propofol and sevoflurane. Data are mean ± SD. *p < 0.05 versus control; ^#^ *p* < 0.05 versus hypoxia.

Based on previous observation that anesthetic improved [Cl^−^]_i_ homeostasis and its close relationship with chloride channels,^15^ we then hypothesized that NKCC1 blocker BTN and KCC2 agonist NEM may help maintain [Cl^−^]_i_ homeostasis following hypoxia. Intracellular [Cl^−^]_i_ levels were determined by the MQAE fluorescence intensities using flow cytometry. We found that hypoxia increased intracellular [Cl^−^]_i_ (indicated by decreased fluorescence intensity), which was unchanged by sub‐dosed combined PS. As expected, both BTN and NEM treatment improved the [Cl^−^]_i_ balance (Figure 2A). We also measured if restoration of [Cl^−^]_i_ balance is associated with improved neuronal survival. Indeed, we observed that both BTN and NEM significantly increased neuronal viability and decreased neuronal death (Figure 2B,C), consistent with the critical role on [Cl^−^]_i_ balance on cell survival upon excitotoxicity.

### 3.3 Both BTN and NEM preserved BDNF and GABA_A_R subunit balance in neuronal cultures

Since hypoxia reduced BDNF expression in hippocampal neuronal cultures (Figure 1C,D), we then detected whether BTN and NEM could preserve BDNF expression. As shown in Figure 2D, significant increased BNDF protein levels were evident after BTN and NEM treatment compared to hypoxia alone. This may participate in neuronal protection by inhibiting excitotoxicity via decreased calcium release.

The Cl^−^ is associated with GABA_A_R remodeling and subunit imbalance. We then explored the effect of BTN and NEM on GABA_A_R subunits in neuronal cultures. Interestingly, hypoxia led to a decrease in GABA_A_R α1 and an increase in GABA_A_R α5 (Figure 2E,F). This imbalance may contribute to GABAergic synaptic dysfunction and decompensated hypoxia‐related excitotoxicity. Importantly, both BTN and NEM helped to restore the GABA_A_R subunit balance (Figure 2E,F), suggesting that restoration of Cl^−^ indeed preserves GABA_A_R structure.

### 3.4 Both BTN and NEM promoted GABAergic synaptic function and memory performance in hypoxic mouse models

**FIGURE 3:**
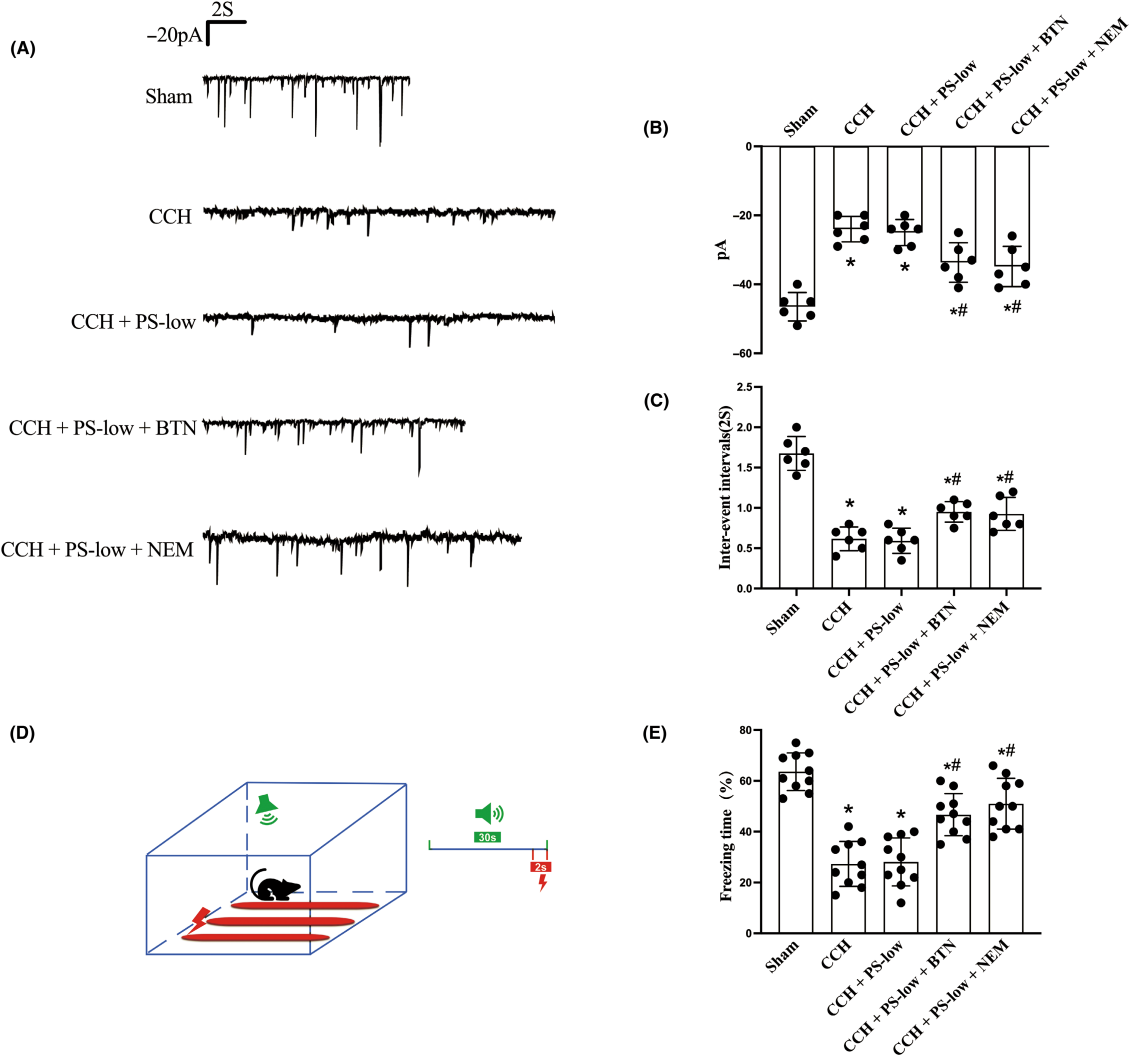
Both BTN and NEM promoted GABAergic synaptic function and memory performance in hypoxic rat models. Aged rats were subjected to CCH, followed by PS‐low, BTN or NEM treatment. Hippocampal slices were subjected to electrophysiologic analysis and contextual memory performances were evaluated. (A) Tracing of mIPSC potential, (B) amplitude and (C) inter‐event interval of the GABA inhibition current. (D) Schematic showing the protocol for contextual memory test. (E) Percentage of freezing time in the contextual memory test. BTN, bumetanide; CCH, chronic cerebral hypoxia; mIPSC, micro‐postsynaptic inhibitory currents; NEM, N‐ethylmaleimide; PS‐low, sub‐anesthetic dose of combined propofol and sevoflurane. Data are mean ± SD. *p < 0.05 versus sham; ^#^ *p* < 0.05 versus CCH.

To validate that the improvement in GABA_A_R subunit balance could promote GABAergic synaptic function in vivo, mIPSC in pyramidal neurons in the CA1 region of the hippocampal slices were recorded, and the amplitude and frequency of the mIPSC potential were measured (Figure 3A). As expected, chronic hypoxia caused a decrease in amplitude and a longer interval between events (IEI), suggesting GABAergic synaptic dysfunction (Figure 3B,C). Administration of BTN or NEM partially reversed this change (Figure 3B,C), indicating a partial restoration of GABA compensatory efficiency against excitotoxicity.

To further test if this restoration GABA compensatory effect is associated with better cognitive function, memory performance in CCH rats was evaluated. We found that BTN and NEM could both ameliorate impaired memory function (Figure 3D,E).

### 3.5 Both BTN and NEM decreased CCH‐induced hippocampal CA1 neuronal loss

**FIGURE 4:**
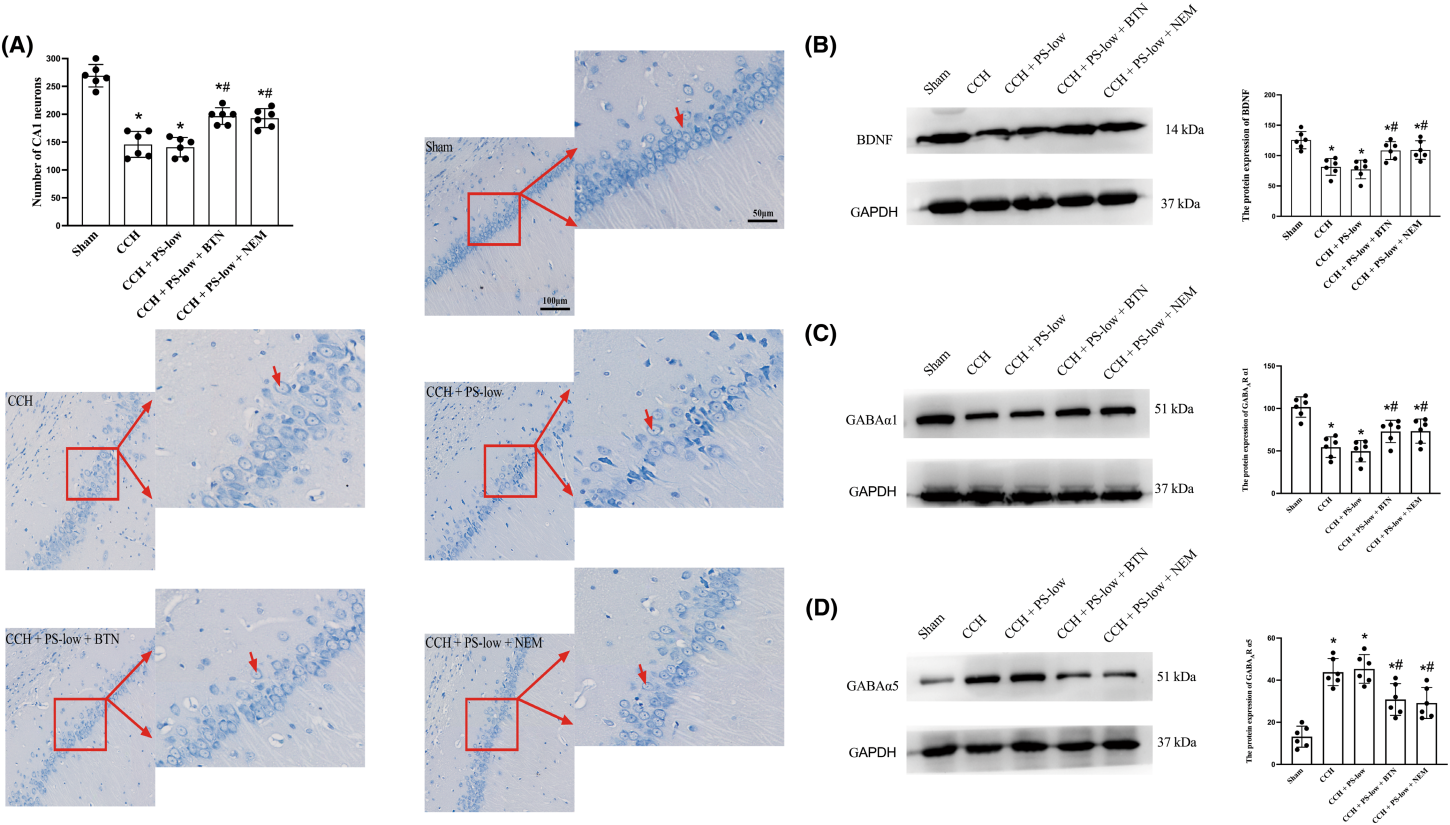
Both BTN and NEM decreased CCH‐induced hippocampal CA1 neuronal loss, preserved the BDNF expression, and prevented GABA_A_R subunit imbalance in CCH brains. Brain slices were subjected to Nissl staining and imaging. (A) Cell counting of the CA1 neurons was performed. (B) Western blotting and semi‐quantification of BDNF levels. (C) Western blotting and semi‐quantification of GABA_A_R α1 levels. (D) Western blotting and semi‐quantification of GABA_A_R α5 levels. BTN, bumetanide; CCH, Chronic cerebral hypoxia; NEM, N‐ethylmaleimide; PS‐low, sub‐anesthetic dose of combined propofol and sevoflurane. Data are mean ± SD. *p < 0.05 versus Sham; ^#^ *p* < 0.05 versus CCH.

Given that the hippocampal neurons play a critical role in cognitive function, we then assessed the neuronal damage in hippocampal CA1 region. Nissl staining was performed on brain slices, and the number of live CA1 neurons was counted. The results showed that CCH caused significant loss of CA1 neurons, which was partially rescued by both BTM and NEM (Figure 4A). This may serve as the mechanism for their effects on cognitive preservation.

### 3.6 Both BTN and NEM preserved the BDNF and prevented GABA_A_R subunit imbalance in CCH brains

Finally, we validated our in vitro findings regarding BTN's and NEM's effects on BDNF and GABA_A_R in CCH models in vivo. Same as in vitro, downregulation of BNDF protein levels was also partially attenuated by both BTN and NEM in vivo (Figure 4B). In addition, both drugs could partially alleviate the subunit imbalance involving GABA_A_R α1 and GABA_A_R α5 (Figure 4C,D).

## 4 DISCUSSION

CCH is a reliable model for VCID, associated with damage to the hippocampus and white matter and ultimately brain atrophy and cognitive dysfunction.^1^, ^30^ Among the many factors that affect cognitive impairment in the perioperative period, the effect of general anesthesia on cognitive impairment is controversial,^31^ making anesthesia in VCID patients particularly challenging. In the present study, anesthetic agents were selected with caution to assure no exacerbation in ischemic–hypoxic injury. Importantly, when combined with [Cl^−^]_i_ regulators, potential protection by anesthetic agents could be further augmented.

Propofol (2,6‐diisopropylphenol) is a widely used intravenous anesthetic worldwide, with average blood concentration for anesthesia maintenance being 4 μg mL^−1^.^32^ Studies have shown that when the average arterial blood concentration of propofol in rats is 4, 1.2, and 0.6 μg mL^−1^, the infusion rate is 40, 20, and 10 mg kg^−1^ h^−1^, respectively.^33^ We previously found that the sub‐anesthetic dose (1.2 μg mL^−1^, 20 mg kg^−1^ h^−1^) of propofol has a neuroprotective effect on rats with cerebral ischemia reperfusion injury,^34^ while high anesthetic dose (4 μg mL^−1^, 40 mg kg^−1^ h^−1^) hinders the neurogenesis and recovery and increases the mortality.^35^ As for sevoflurane, its MAC in adult rats is 2.3% ± 0.3%, and the 99% effective dose is 1.3 MAC.^36^ Previous reports showed that 1.3 MAC sevoflurane has a negative effect on cognitive function, which was absent with 0.65 MAC.^37^, ^38^ In the present study, 1.2 μg mL^−1^ propofol combined with 0.7 MAC sevoflurane was employed as sub‐dose combined anesthesia, affording a proper depth of clinical anesthesia without affecting cognitive function.^39^

The accumulation of extracellular excitatory amino acids caused by cerebral hypoxia–ischemia leads to neuronal excitotoxicity and eventually cell apoptosis in VCID.^40^, ^41^ The hippocampal slices^42^ and neurons^43^ of rats proved that ischemia can induce an immediate increase in intracellular [Cl^−^]_i_. Loss of GABA‐mediated inhibition may participate in the process of overexcitement of neurons.^44^ This present study also confirmed the damage caused by ischemia–hypoxia to neurons (Figures 2 and 4). Many studies have confirmed that BDNF protects against ischemia–hypoxic injury,^45^, ^46^ partially by controlling the ion channels.^47^ For example, the regulation of NKCC1 and KCC2 function by BDNF–TrkB signaling during neuronal development has been widely accepted,^48^, ^49^ and re‐establishing E/I homeostasis provided therapeutic potential against neurodevelopmental disorders. Since increased BDNF reduced excitotoxicity through inhibiting the excessive release of Ca^2+,^ ^18^ we were curious about its relationship with intracellular [Cl^−^]_i_. Surprisingly, regulating [Cl^−^]_i_ did restore BDNF expression, although detailed mechanism needs further exploration. This accidental discovery emphasizes the concept that regulating NKCC1/KCC2 can augment various protective effects.

Intracellular Cl^−^ is strictly controlled by NKCC1‐mediated influx and KCC2‐mediated efflux. Previous studies have found that early treatment of BTN, an FDA‐approved NKCC1 inhibitor, can attenuate hippocampal memory impairment caused by hypoxia–ischemia.^50^ Similarly, BTN also reversed AD‐related behavioral dysfunction by inhibiting neuronal hyperexcitability in aged APOE4 knock‐in mice.^51^ On the other hand, our group reported that propofol‐afforded protection was abolished when the expression or activity of KCC2 protein was inhibited.^15^, ^16^ Therefore, our results proved the presence of an NKCC1/KCC2− [Cl^−^]_i_ ‐GABA_A_R pathway, which is critical for compensating hypoxic brain damage. Interestingly, GABA_A_R activation is also shown to facilitate the stability of KCC2 on the plasma membrane,^52^ suggesting [Cl^−^]_i_ as an important intracellular messenger that participates in a complex interaction between GABA_A_R and KCC2. The specific mechanism may be related to the change in pH caused by altering the concentration of [Cl^−^]_i_, which leads to the conformational changes in GABA synapse.^53^

The drug selection and dosage of this experiment are based on previous publications combined with our observation. We did not monitor or titrate effective brain drug concentration, which is a shortcoming of this study. Due to sexual dimorphism in the mitochondrial metabolic protein profiles,^54^ cerebral perfusion trajectories,^55^ and gene expression of cerebral microvessels,^56^ female rats will be included in future studies. Most of the previous reports only confirmed the effectiveness of [Cl^−^]_i_ modulators, but lacked long‐term observations. In addition, how [Cl^−^]_i_ alterations lead to changes in GABA_A_R subunits and BDNF expression remains unclear. In the present study, we proved the protective effects of sub‐dose anesthetics combined with [Cl^−^]_i_ regulators, although the underlying mechanisms are still unknown. With the development of medical care, anesthesiologists have stepped out of the operating room looking for cooperation with clinical departments. The perioperative period should not only focus on protecting the patient's life but also take various measures to improve the patient's physical condition. This is a radical change and requires a lot of work and effort from all anesthesiologists.

In summary, we first proved that low dose of propofol and sevoflurane is superior to high dose in terms of neuronal protection against ischemic injury. On top of that, the addition of BTN or NEM reversed the CCH‐mediated [Cl^−^]_i_ imbalance, effectively rescued neurons, restored GABAergic systems, and protected the cognitive function in VCID.

## AUTHOR CONTRIBUTIONS

Study concept and design: HW, CY, and YW. Experimental studies: CY and YW. Acquisition, analysis, or interpretation of data: CY and YW. Drafting of the manuscript: CY and YW. Critical revision of the manuscript for important intellectual content: All authors. Statistical analysis: CY and YW. Obtained funding: HW and CY. Administrative, technical, or material support: YL, XW, WH, and ZY. Study supervision: HW.

## CONFLICT OF INTEREST STATEMENT

The authors declare that the research was conducted independent of any commercial or financial relationships that could be construed as a potential conflict of interest.

## Supporting information


Figure S1

## Data Availability

The data that support the findings of this study are available from the corresponding author upon reasonable request.
