# Supplementary material for: Sub‐dose anesthetics combined with chloride regulators protect the brain against chronic ischemia–hypoxia injury
Source: CNS Neurosci Ther. 2023 Aug 6;30(2):e14379. doi: 10.1111/cns.14379 (PMC10848060; doi:10.1111/cns.14379)
Supplement: Supplementary file 1 — Figure S1 [file CNS-30-e14379-s001.docx]

**
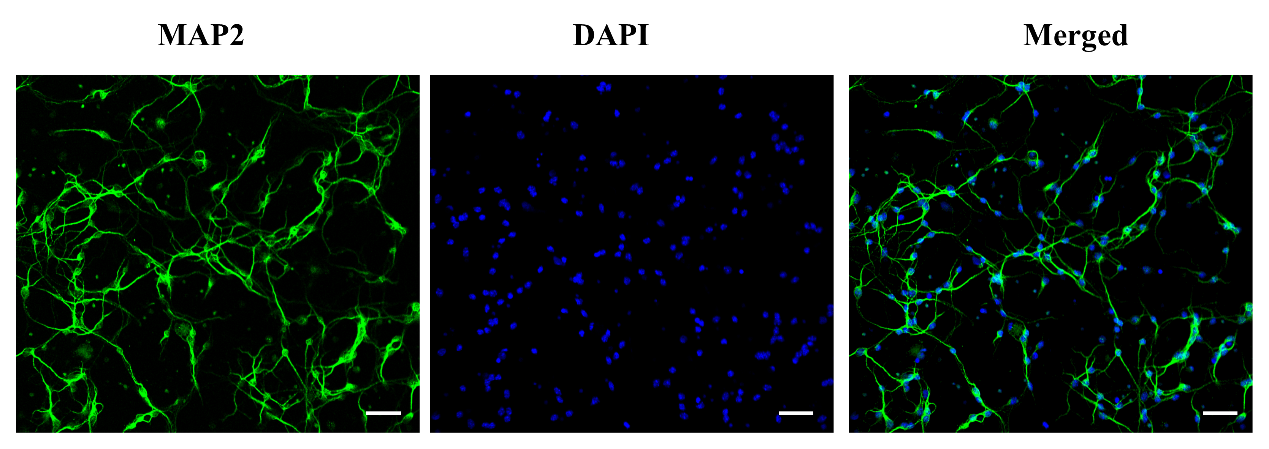
**

**Figure S.** **Identification of cultured primary hippocampal neurons.**

Microtubule-associated protein 2 (MAP2) and 4,6-diamidino-2-phenylindole (DAPI) were used to identify the purity of cultured primary hippocampal neurons by immunofluorescence. The percentage of cultured neurons was above 96 ±3.27%. Scale bars = 50 μm

**Immunofluorescence assay**

To determine the extracted hippocampal neurons purity, the primary and secondary antibodies used were anti-MAP-2 (1:1,000; Abcam Cat# ab32454, RRID:AB776174) and goat anti-rabbit IgG H&L (Alexa Fluor® 488) (1:1,000 for immunofluorescence; Abcam Cat# ab150077, RRID:AB2630356), respectively. A Zeiss LSM 510 Meta confocal system equipped with 10× and 40× objectives was used to observe neurons, and a 488 nm argon laser was used to excite the fluorophores. All experiments were repeated three times independently.
